# Where you live matters, but action is personal: insights from multilevel analysis of pre-exposure prophylaxis awareness, willingness, and use among men who have sex with men in China

**DOI:** 10.1186/s40249-026-01462-5

**Published:** 2026-05-25

**Authors:** Nahom Kiros Gebregziabher, Junfeng Zhang, Qinghua Shang, Qingcui Wu, Zhaoyu Cheng, Hui Gong, Maohe Yu, Zhongdan Chen, Changping Li, Zhuang Cui

**Affiliations:** 1 Department of Epidemiology and Biostatistics, School of Public Health, Tianjin Medical University, No. 22 Qixiangtai Road, Heping District, Tianjin, 300070 China; 2 Tianjin Key Laboratory of Environment, Nutrition and Public Health, Tianjin, China; 3 Department of Public Health, Second Hospital of Tianjin Medical University, Tianjin, 300211 China; 4 STD & AIDS Control and Prevention Section, Tianjin Center for Disease Control and Prevention, Tianjin, China; 5 Tianjin Key Laboratory of Pathogenic Microbiology of Infectious Disease, Tianjin Centers for Disease Control and Prevention, Tianjin, 300011 China; 6 HIV/Hepatitis/STI/TB, World Health Organization Representative Office in China, 401 Dongwai Diplomatic Building 23, Dongzhimenwai Dajie, Chaoyang District, Beijing, 100600 China

**Keywords:** HIV, AIDS, Men who have sex with men, Pre-exposure prophylaxis, Andersen’s behavioral model, Multi-level determinants, Prevention, Health equity, Infectious disease

## Abstract

**Background:**

Pre-exposure prophylaxis (PrEP) is a highly effective HIV prevention strategy, yet its uptake among men who have sex with men (MSM) in China remains low. This study aimed to assess whether city-level differences influence the PrEP awareness, willingness, and ever-use among MSM in China.

**Methods:**

A secondary analysis of a national cross-sectional survey of MSM (*n* = 5041) from 42 Chinese cities was conducted. We fitted generalized linear mixed models with city as a random intercept for awareness and ever-use. Willingness showed no significant clustering by city and was analyzed using logistic regression. Independent variables were grouped according to Andersen’s Behavioral Model of Health Services Use into predisposing, enabling, and need-related factors. Interclass correlation (ICC) quantifies the variance between cities.

**Results:**

The majority (87.2%) had heard of PrEP, 58.4% were willing to use it, and 13.0% had ever used it. In fully adjusted models, city-level clustering remained evident for PrEP awareness (ICC = 5.3%, *P* = 0.003) and ever-use (ICC = 8.1%, *P* = 0.011), indicating a measurable influence of place of residence. Willingness showed no significant between-city variation. PrEP awareness was higher among younger MSM (a*OR* = 0.98, *P* < 0.05) and those with lower income (< 3000 CNY: a*OR* = 0.67, *P* < 0.05), and not knowing partners’ HIV status (a*OR* = 0.57, *P* < 0.001) was associated with reduced awareness. Willingness to use PrEP was lower among those earning < 3000 CNY (a*OR* = 0.75, *P* < 0.05), positively associated with higher per capita health expenditure (a*OR* = 1.02, *P* < 0.05), and negatively associated with higher HIV prevalence (a*OR* = 0.96, *P* < 0.01). Ever use of PrEP was associated with condomless sex (a*OR* = 1.62, *P* < 0.001), while lower income (< 3000 CNY: a*OR* = 0.45, *P* < 0.001) remained a significant barrier.

**Conclusions:**

Place of residence affects PrEP awareness and use, but not willingness. However, actual use is primarily driven by individual-level factors. HIV prevention in China should combine city-level service expansion with targeted individual-level interventions to close the gap between PrEP awareness, willingness, and actual use.

**Graphical abstract:**

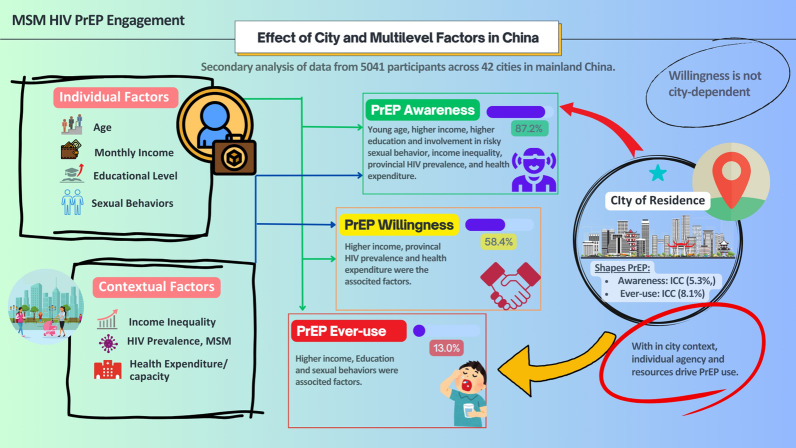

**Supplementary Information:**

The online version contains supplementary material available at 10.1186/s40249-026-01462-5.

## Background

The global fight against HIV/AIDS has made remarkable progress over the past decades. Despite the observed success rates in the general population, certain groups, including sex workers, intravenous drug users, and men who have sex with men (MSM), continue to face disproportionately high infection rates [1, 2]. In fighting HIV, China’s HIV/AIDS policy evolved from a narrow health-focused approach in the 1980 s to a coordinated, multi-sectoral strategy by the 2000 s, followed by the 2004 creation of the State Council AIDS Working Committee [3]. In China, the MSM accounted for approximately 23.0% of new HIV diagnoses in recent years [4], with prevalence varying by geographical location [5]. Incidence rates among the MSM were also found to vary across different cities. For example, in a study conducted in Sichuan province, the estimated HIV-1 incidence (2018 to 2022) among MSM was 4.6% in Chengdu, 1.2% in Dazhou, 1.2% in Guangyuan, and 10.7% in Luzhou [6]. This trend underscores persistent gaps in HIV prevention and service delivery for this key population across cities.

Pre-exposure prophylaxis (PrEP), a biomedical intervention involving daily oral antiretrovirals, has emerged as a highly effective tool for preventing HIV acquisition. When taken consistently, PrEP can reduce the risk of HIV infection by over 90% among MSM [7]. PrEP was started in China as a pilot project in 2017 [8]. Currently, PrEP uptake in China remains low, with evidence showing disparities in awareness, willingness, and use across regions within the country [9]. Barriers, such as low awareness, concerns about stigma, the cost of medication, a lack of provider engagement, and limited promotion, contribute to this disconnect [10, 11]. The observed disparities in accessing PrEP services across different cities, as reported in several studies [12, 13], suggest that both individual and contextual factors must be considered to comprehensively understand the phenomenon.

Despite growing evidence on PrEP acceptability, little is known about how multilevel contextual and individual factors jointly influence PrEP engagement among MSM in China. Most of the existing literature in China discusses the use of PrEP-related services among MSM from an individual or structural perspective separately. MSM is still a stigmatized, hidden population in China [14], although the relative acceptance seems to be increasing over time. Some major cities, such as Chengdu, were reportedly more open than other cities in China [15]. Such differences may affect the dynamics of MSM interaction in different cities, forming their own subculture in which members interact based on the context in which they live. This was reflected in part by a spatiotemporal analysis of MSM in China, which showed a diverse social app usage pattern across the cities and provinces in China [16]. Such unmeasured factors might, in turn, be affected by macro-level factors that can differ from place to place.

In this study, we employed Andersen’s Behavioral Model of Health Services Use [17], a widely adopted theoretical framework that explains health-seeking behavior through three primary domains. This model recognizes that determinants of health services use operate at both the individual and contextual levels [17]. While this model has been applied in several HIV-related studies globally, its use in the Chinese context, particularly in relation to PrEP uptake among MSM, remains limited. This study applies Andersen’s Behavioral Model within a multilevel analytic framework to disentangle individual and contextual influences on PrEP awareness, willingness, and use, a perspective that has not been previously examined in China. More specifically, the study aims to quantify the extent to which city-level clustering contributes to the variance in awareness, willingness, and use of PrEP.

## Methods

### Study design and setting

This study is a secondary analysis of a national cross-sectional survey of MSM in China. The original survey aimed to assess awareness, attitudes, and service needs related to PrEP. The data was collected between October 20 and December 20, 2021, covering 31 Chinese regions, comprising 22 provinces, four municipalities, and five autonomous regions. The recruitment involved both online and offline methods, employing convenience and snowball sampling. MSM community organizations participated in the online distribution of electronic questionnaires and in the recruitment of participants via WeChat Moments and Groups. In the offline approach, Shenlan Public Health Counseling Service Center staff members visit establishments frequented by the MSM to recruit participants. This way, a total of 6535 MSM who self-reported to have been engaging in sexual intercourse with at least one man during the six months preceding the data collection time were recruited. The sample comprised MSM across 258 cities in China. The first requirement to start HIV PrEP is to be free from HIV. Hence, for the current analysis, participants who did not report their HIV status (*n* = 485) were excluded. To ensure statistically meaningful comparisons and robust multilevel modeling, cities with at least 30 participants were retained for the current analysis. This yielded 5041 HIV negative participants from 42 cities across all major regions.

### Theoretical framework

Andersen’s Behavioral Model of Health Services Use conceptualizes health service utilization as being shaped by three primary domains, namely predisposing, enabling, and need factors [18]. Predisposing factors refer to pre-existing individual characteristics that influence a person’s inclination or motivation to engage in a health behavior, independent of immediate resource availability. These typically include demographic attributes (e.g., age), social structure indicators (e.g., education, occupation), and aspects of identity or behavioral orientation that may shape health beliefs and awareness. Enabling factors represent the structural, economic, and contextual conditions that facilitate or constrain an individual’s ability to access and utilize health services. These include both personal resources (e.g., income, access to information) and broader environmental characteristics (e.g., healthcare infrastructure, regional socioeconomic conditions). Need factors refer to the perceived or clinically evaluated necessity for a health intervention, reflecting an individual’s risk profile or health status. These factors are often the most immediate drivers of health behavior, as they relate to perceived vulnerability or objective risk. In the current study, these domains were operationalized at both the individual and contextual levels, enabling a multilevel understanding of PrEP-related behaviors among MSM.

### Outcome variables

PrEP awareness was measured as whether the participant had ever heard of HIV PrEP before. Willingness to use PrEP means whether the participant expressed willingness to use HIV PrEP in the future, without conditions or qualifiers regarding cost or anything else. PrEP use was measured as whether a participant had ever used HIV PrEP regardless of their current use status.

### Independent variables

Individual-level independent variables captured socio-demographic and behavioral characteristics of MSM participants. Predisposing factors reflect individual background. Enabling factors represent resources available to access prevention services. Need-related factors directly indicate HIV risk exposure. Predisposing contextual factors describe the broader socio-economic environment. Enabling factors measure the capacity and investment in health services. The Need-related contextual variable approximates the local HIV burden among the MSM (Table 1). For this study, contextual-level aggregate data were collected from several sources. The provincial HIV prevalence estimate was obtained from a large-scale systematic review and meta-analysis [5]. The Greater income inequality (GINI) index for 2021 was sourced from an article published in 2024 [19]. The other contextual variables were collected from the National Bureau of Statistics of China and the China Statistical Yearbook for 2021, which is available online [20]. These macro-level factors, measured at the provincial level, served as proxies for city-level contextual factors because city-level data were unavailable. Hence, they should be interpreted as reflecting broader regional estimates rather than city-specific.

**Table 1:** Independent variables by level and domain

Level	Domain	Variable
Individual	Predisposing	Age
		Education level
		Occupation
	Enabling	Monthly income in Chinese Yuan (CNY)
	Need-related	Sex role
		History of commercial sex
		Knowledge of partners’ HIV status
		Condom use
		Group sex, number of sexual partners
Contextual	Predisposing	Income inequality
		Urbanization rate
	Enabling	Health professionals per capita
		Per capita healthcare expenditure
	Need-related	Provincial HIV prevalence among MSM

### Statistical analysis

Descriptive statistics, such as means and standard deviations, frequencies, and proportions, were used to summarize the overall sample characteristics and for each PrEP outcome. To identify the determinants of PrEP awareness and use, generalized linear mixed models (GLMM) with a logit link function and random intercepts for cities were employed. This accounted for clustering at the city level. The reference category for each binary outcome was “No” (i.e., 0 = unaware or never used). Four models were planned for each outcome: (1) an unconditional (null) model to estimate city-level variance; (2) a model including only individual-level predictors; (3) a contextual-level model; and (4) a full model combining individual and contextual variables. After the unconditional model, a bivariate analysis was conducted, and variables that had a significant level of < 0.05 were selected for the subsequent models. The intraclass correlation coefficient (ICC) was computed to assess the proportion of total variance attributable to city-level clustering using the formula: [ICC = τ02/τ02 + σ2], where τ02 represents the variance of the random intercept, and σ2 is the level-1 residual variance (set to π^2^/3 in logistic models). Model fit was assessed using the Akaike Information Criterion (AIC) and the Bayesian Information Criterion (BIC). For the willingness to use PrEP outcome, multilevel modeling was not used due to a non-significant ICC in the unconditional model. Therefore, binary logistic regression was applied using the same set of predictors. The Gini index was included as a proportion (ranging from 0 to 1), and the reported odds ratios correspond to a one-unit increase on this scale. Healthcare expenditure was measured in CNY per capita. The regression models are expressed in increments of 1000 CNY, so the odds ratios reflect the effect of each 1000-CNY increase. All analyses were conducted using IBM SPSS Statistics for Windows, version 28 (IBM Corp., Armonk, N.Y., USA), with statistical significance set at *P* < 0.05. The radar chart was generated using Microsoft Excel 16.108 (Microsoft Corporation, Redmond, WA, USA). R software, version 4.5.2 (R Foundation for Statistical Computing, Vienna, Austria), was used to produce the forest plots. Results are presented in summary tables and forest plots, with additional details in the supplementary material.

## Results

This study assesses the individual and contextual factors associated with PrEP engagement among MSM in China. Table 1 presents the background characteristics of the study population across the three outcomes. Group-wise characteristics are reported only for participants who responded “yes” to each outcome. The overall mean age of the participants was 29.7 ± 8.1. The majority (92.9%) of MSM with postgraduate education reported having heard of PrEP, compared to only 75.8% among those with junior high or lower education (*P* < 0.001). Differences by employment type were also evident, with students and those not in the labor force reporting higher proportions of awareness. Higher-income participants (> 8000 CNY/month) had the highest proportions of awareness (91.1%) and use (18.7%), compared to lower-income groups (*P* < 0.001). Willingness to use PrEP showed a small difference in proportions across the income categories, an enabling factor. PrEP use has a relatively large difference in proportion between those reporting a history of commercial sex and those who did not (Table 2).

**Table 2:** Background characteristics of study participants overall and by each PrEP outcome

Variable	Category	Total (*n* = 5041), *n* (%)	Heard about PrEP (*n* = 4396), *n* (%)	*P*-value	Willing to use (*n* = 2217) PrEP, *n* (%)	*P*-value	Ever used PrEP (*n* = 570), *n* (%)	*P*-value
Education level	Junior high & below	302 (6.0)	229 (75.8)	< 0.001	137 (59.6)	0.21	28 (12.2)	< 0.001
	High school/Tech sec	812 (16.1)	681 (83.9)		331 (54.5)		65 (9.5)	
	College/Undergrad	3366 (66.8)	2965 (88.1)		1506 (58.9)		380 (12.8)	
	Postgraduate or above	561 (11.1)	521 (92.9)		243 (59.7)		97 (18.6)	
Occupation type	Not in the labor force	150 (3.0)	135 (90.0)	< 0.001	65 (58.6)	0.54	20 (14.8)	0.001
	Student	857 (17.0)	773 (90.2)		375 (56.9)		75 (9.7)	
	Informal/self-employment	1773 (35.2)	1494 (84.3)		803 (59.9)		175 (11.7)	
	Formal employment	2261 (44.9)	1994 (88.2)		974 (57.7)		300 (15.0)	
Monthly income	No stable income	938 (18.6)	833 (88.8)	< 0.001	410 (56.9)	0.13	81 (9.7)	< 0.001
	< 3000 CNY	450 (8.9)	380 (84.4)		179 (52.5)		43 (11.3)	
	3000–5000 CNY	1308 (25.9)	1086 (83.0)		585 (59.9)		116 (10.7)	
	5000–8000 CNY	1184 (23.5)	1039 (87.8)		519 (59.4)		132 (12.7)	
	> 8000 CNY	1161 (23.0)	1058 (91.1)		524 (59.1)		198 (18.7)	
Commercial sex, ever	No	4755 (94.3)	4139 (87.0)	0.16	2089 (58.3)	0.65	510 (12.3)	< 0.001
	Yes	286 (5.7)	257 (89.9)		128 (59.8)		60 (23.3)	
STI (past one year)	No	4621 (91.7)	4027 (87.1)	0.67	2045 (58.5)	0.51	500 (12.4)	< 0.001
	Yes	420 (8.3)	369 (87.9)		172 (56.6)		70 (19.0)	
Number of sex partners (past 6 months)	1–5	4364 (86.6)	3810 (87.3)	0.54	1922 (58.3)	0.93	430 (11.3)	< 0.001
	6–10	446 (8.8)	390 (87.4)		187 (58.4)		73 (18.7)	
	> 10	231 (4.6)	196 (84.8)		108 (59.7)		67 (34.2)	
Group sex (past 6 months)	No	4383 (86.9)	3812 (87.0)	0.21	1926 (58.3)	0.83	417 (10.9)	< 0.001
	Yes	658 (13.1)	584 (88.8)		291 (58.8)		153 (26.2)	
Sex role	Receptive	1617 (32.1)	1443 (89.2)	< 0.001	705 (56.5)	0.34	204 (14.1)	0.001
	Versatile	1148 (22.8)	1011 (88.1)		496 (58.1)		154 (15.2)	
	Insertive	1924 (38.2)	1653 (85.9)		863 (59.9)		190 (11.5)	
	Oral only	352 (7.0)	289 (82.1)		153 (59.5)		22 (7.6)	
Condom use at last anal sex	No	931 (18.5)	809 (86.9)	0.47	412 (58.1)	0.92	158 (19.5)	< 0.001
	Yes	3758 (74.5)	3298 (87.8)		1652 (58.3)		390 (11.8)	
Partner’s HIV status (past 6 months)	Didn’t know all	868 (17.2)	702 (80.9)	< 0.001	383 (57.9)	0.31	92 (13.1)	< 0.001
	Partially knew	1800 (35.7)	1599 (88.8)		813 (60.0)		251 (15.7)	
	Knew all	2373 (47.1)	2095 (88.3)		1021 (57.3)		227 (10.8)	

The table presents data on those who are aware of PrEP, are willing to use it in the future, and have ever used it. The full table is in the supplementary material. *STI* sexually transmitted infection

### Factors associated with awareness of HIV PrEP

**Fig. 1:**
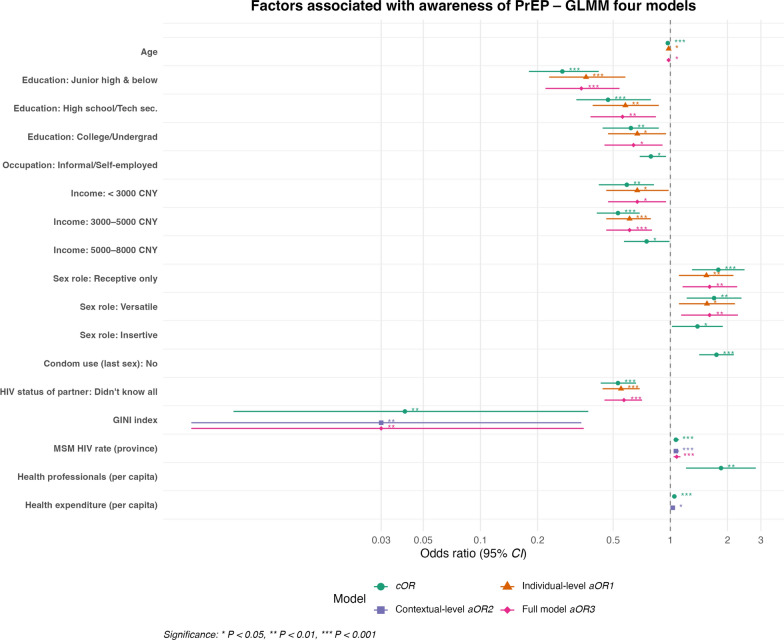
Multilevel logistic regression analysis of factors associated with PrEP awareness. The vertical dashed line indicates the null value (*OR* = 1). Estimates to the right of the line indicate higher odds of PrEP awareness, while those to the left indicate lower odds. Models account for clustering at the city level using a GLMM. *cOR* crude odds ratio, *aOR* adjusted odds ratio. Reference categories: Postgraduate or above; Formal Employment; > 8000 CNY; Oral only role; Knew all partners’ HIV status

In the final model, several predisposing factors were significantly associated with PrEP awareness. Age showed a modest but consistent inverse association (*aOR* = 0.98; 95% *CI*: 0.97–0.99; *P* < 0.05), indicating that younger MSM were more likely to have heard of PrEP. MSM with junior high education or below had 66% lower odds of awareness compared to those with graduate-level education (*aOR* = 0.34; 95% *CI*: 0.22–0.54; *P* < 0.001), while those with only high school or technical education also showed reduced awareness (*aOR* = 0.56; 95% *CI*: 0.38–0.84; *P* < 0.01), as compared to the higher educational category. College/undergraduate education remained marginally significant (*aOR* = 0.64; 95% *CI*: 0.45–0.91; *P* < 0.05). MSM who identified as receptive (*aOR* = 1.61; 95% *CI*: 1.16–2.25; *P* < 0.01) or versatile (*aOR* = 1.61; 95% *CI*: 1.14–2.27; *P* < 0.01) had significantly higher odds of awareness than their oral-only counterparts. As enabling factors, those earning less than 3000 CNY/month had 33% lower odds of being aware compared to those earning more than 8000 CNY (*aOR* = 0.67; 95% *CI*: 0.47–0.95; *P* < 0.05), and those earning between 3000 and 5000 CNY had similar reduced odds of awareness (*aOR* = 0.61; 95% *CI*: 0.46–0.80; *P* < 0.001). Among need-related factors, not knowing the HIV status of all sexual partners significantly lowered the odds of PrEP awareness (*aOR* = 0.57; 95% *CI*: 0.45–0.71; *P* < 0.001). The GINI index, a contextual-level predisposing factor, was negatively associated with awareness (*aOR* = 0.03; 95% *CI*: 0.003–0.35; *P* < 0.01). Although per capita health expenditure, an enabling factor, showed a positive association with awareness in the contextual-only model (*aOR* = 1.03; 95% *CI*: 1.01–1.06; *P* < 0.05), this effect diminished in the final model. As a contextual need factor, provincial HIV prevalence was strongly associated with increased awareness (*aOR* = 1.08; 95% *CI*: 1.04–1.13; *P* < 0.001) (Fig. 1).

### Variance components and model fit for HIV PrEP awareness

For this outcome, four hierarchical models were constructed. The ICC for the unconditional model was 6.7% (95% *CI*: 3.8%–11.6%), indicating a modest level of between-city clustering in PrEP awareness. When individual-level predictors were added, ICC dropped slightly to 5.4%, suggesting compositional effects of individual demographics. The contextual-only model showed little change in ICC (6.69%), indicating that the measured structural factors did not meaningfully explain the city-level variance. In the full model, ICC was 5.3% (95% *CI*: 2.8%–9.7%). Model fit statistics (AIC and BIC) indicated that the unconditional model had the lowest AIC (25,595). However, the final model provided a more conceptually comprehensive understanding of PrEP awareness, despite having a higher AIC (26,255) (Table 3).

**Table 3:** Random-intercept estimates and ICCs across models for PrEP awareness

	Unconditional model	Individual-level model	Contextual-level model	Final model (full)
City-Level Variance (Ʈb)	0.236	0.186	0.237	0.183
Standard Error	0.073	0.062	0.074	0.062
Z-value	3.239	2.993	3.223	2.967
*P*-value	0.001	0.003	0.001	0.003
ICC (%) [95% *CI*]	6.7% [3.8%–11.6%]	5.4% [2.9%–9.8%]	6.69% [3.8%–11.7%]	5.3% [2.8%–9.7%]
Fit Statistics				
AIC	25,595	26,012	25,876	26,255
BIC	25,602	26,019	25,882	26,262

Ʈb = city‑level variance; ICC = intraclass correlation coefficient (proportion of variance due to between‑city differences). *AIC* Akaike Information Criterion; *BIC* Bayesian Information Criterion. Lower AIC/BIC indicates a better fit. Significant Ʈb (*P* ≤ 0.003) supports multilevel modeling

### Factors associated with ever-use of HIV PrEP

**Fig. 2:**
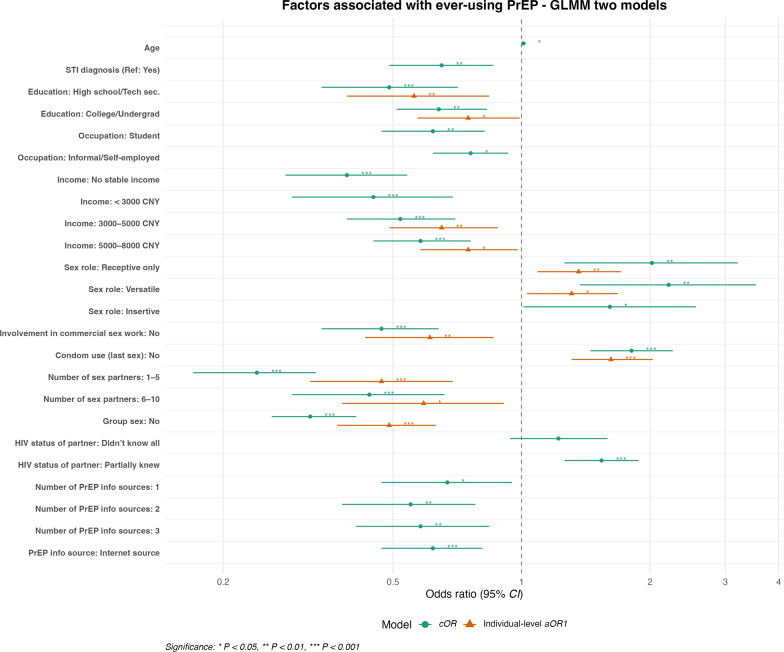
Multilevel logistic regression analysis of factors associated with PrEP ever-use. The vertical dashed line indicates the null value (*OR* = 1). Estimates to the right of the line indicate higher odds of PrEP awareness, while those to the left indicate lower odds. Models account for clustering at the city level using a GLMM. *cOR* crude odds ratio, *aOR* adjusted odds ratio. Reference categories: Postgraduate or above; Formal Employment; > 8000 CNY; Oral only role; Knew all partners’ HIV status; > 10 sexual partners; ≥ 4 sources of PrEP information; Formal PrEP information source

In the final mode, among the predisposing factors, MSM with only high school or technical secondary education have significantly lower odds of PrEP use than those with graduate education or above (*aOR* = 0.56, 95% *CI*: 0.39–0.84, *P* < 0.01). MSM who identified as receptive only (*aOR* = 1.36, 95% *CI*: 1.09–1.71, *P* < 0.01) or versatile (*aOR* = 1.31, 95% *CI*: 1.03–1.68, *P* < 0.05) were significantly more likely to have used PrEP compared to those with an oral-only role. MSM earning less than 3000 CNY had significantly lower odds of PrEP use compared to those earning over 8000 CNY (*aOR* = 0.45, 95% *CI*: 0.29–0.69, *P* < 0.001), and similarly lower odds were observed among those earning 3000–5000 CNY (*aOR* = 0.65, 95% *CI*: 0.49–0.88, *P* < 0.01) and 5000–8000 CNY (*aOR* = 0.75, 95% *CI*: 0.58–0.98, *P* < 0.05). MSM who did not use a condom at last sex had significantly higher odds of using PrEP (*aOR* = 1.62, 95% *CI*: 1.31–2.03, *P* < 0.001). Participants who had 1–5 sexual partners had reduced odds compared to those with more than 10 (*aOR* = 0.47, 95% *CI*: 0.32–0.69, *P* < 0.001), and similarly lower odds were found among those with 6–10 partners (*aOR* = 0.59, 95% *CI*: 0.38–0.91, *P* < 0.05). MSM who had not engaged in group sex had significantly lower odds of PrEP use (*aOR* = 0.49, 95% *CI*: 0.37–0.63, *P* < 0.001). Those not involved in commercial sex work were less likely to have used PrEP (*aOR* = 0.61, 95% *CI*: 0.43–0.86, *P* < 0.01). Although contextual factors were assessed, they were not found to be significantly associated with PrEP use. Hence, for PrEP use, only two models were required, as the second model was also the final model (Fig. 2).

### Variance components and model fit for ever-use of HIV PrEP

The unconditional model showed significant between-city variance in PrEP ever-use, with an ICC of 9.1% (95% *CI*: 5.2%–15.6%), indicating substantial clustering at the city level. When individual-level variables were included, the city-level variance decreased to 0.197 (*SE* = 0.077, *P* = 0.011), and the ICC reduced slightly to 8.1% (95% *CI*: 4.5%–14.3%). Model fit improved with the inclusion of individual-level variables, as indicated by a reduction in AIC (from 22,343 to 21,462) and BIC (from 22,350 to 21,468) (Table 4).

**Table 4:** Random-intercept estimates and ICCs across models for PrEP ever-use

	Unconditional Model	Individual-Level Model (Final)
Random Intercept Estimate (Ʈb)	0.33	0.197
Standard Error	0.102	0.077
Z-value	3.219	2.558
*P* -value	0.001	0.011
ICC (%) [95% *CI*]	9.1% [5.2%–15.6%]	8.1% [4.5%–14.3%]
Fit Statistics		
AIC	22,343	21,462
BIC	22,350	21,468

Ʈb = city‑level variance; ICC = intraclass correlation coefficient (proportion of variance due to between‑city differences). *AIC* Akaike Information Criterion; *BIC* Bayesian Information Criterion. Lower AIC/BIC indicates a better fit. Significant Ʈb (*P* ≤ 0.011) supports multilevel modeling

### Factors associated with willingness to use HIV PrEP

**Fig. 3:**
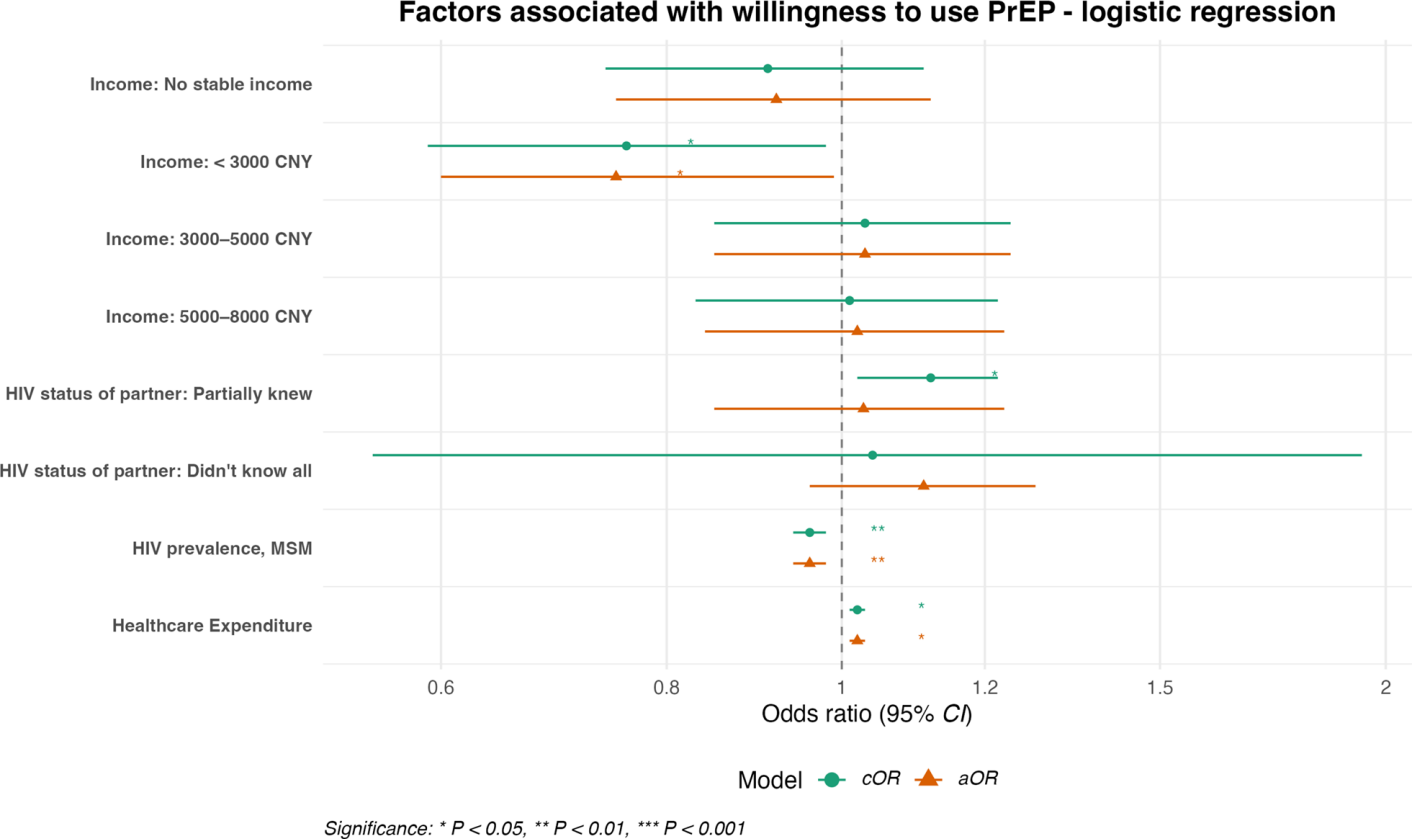
Determinants of willingness to use PrEP: logistic regression analysis. Adjusted odds ratios (*aORs*) and 95% confidence intervals (*CIs*) from logistic regression models assessing determinants of willingness to use PrEP. The vertical dashed line indicates the null value (*OR* = 1). Estimates to the right of the line indicate higher odds of PrEP willingness, while those to the left indicate lower odds. *cOR* crude odds ratio. Reference categories: > 8000 CNY; Knew all partners’ HIV status

Due to the non-significant variance across cities in the unconditional model, multilevel modeling was not warranted for this outcome. Therefore, a binary logistic regression was conducted. Among individual-level enabling factors, monthly income showed a limited but notable association with willingness. Participants earning less than 3000 CNY per month had significantly lower odds of being willing to use PrEP compared to those earning more than 8000 CNY (*aOR* = 0.75, 95% *CI*: 0.60–0.99, *P* = 0.047). At the contextual level, both enabling and need-related factors were significant. Higher per capita healthcare expenditure (a contextual-level enabling factor) was positively associated with the willingness to use PrEP (*aOR* = 1.02, 95% *CI*: 1.01–1.03, *P* = 0.033). Conversely, exposure to high HIV prevalence hotspots, an evaluated need, was negatively associated with willingness (*aOR* = 0.96, 95% *CI*: 0.94–0.98, *P* = 0.004) (Fig. 3).

**Fig. 4:**
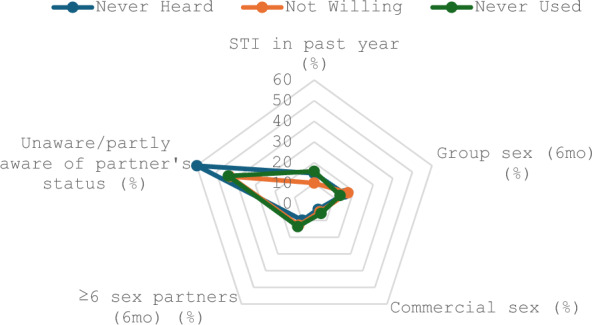
Distribution of HIV risk behaviors among participants reporting condomless anal sex, by PrEP awareness, willingness, and use. Percentages represent high-risk sexual behaviors among participants reporting condomless anal sex who had never heard of PrEP, were not willing to use PrEP, or had never used PrEP, highlighting gaps in prevention among high-risk groups. *STI* sexually transmitted infection, 12 months prior to data collection

As an exploratory extension of the primary analyses, participants who reported condomless anal sex and were unaware of PrEP, were not willing to use, and had never used PrEP, and their involvement in risky behaviors was assessed. Among those who had never heard of PrEP, a striking 59.8% were either unaware or only partially aware of their partners’ HIV status, the highest across all groups. This group also showed notable levels of group sex participation (15.6%) and STI history in the past year (14.8%). Participants not willing to use PrEP demonstrated the highest engagement in group sex (17.2%) and commercial sex (4.7%), while 12.8% reported having six or more male sexual partners in the past six months. Among those who had never used PrEP, 13.6% reported having six or more recent partners, and 15.8% had a recent STI, the highest rates among the three groups (Fig. 4).

## Discussion

This large, multi-city analysis examined the effect of city of residence on HIV PrEP engagement among MSM in China. The modest but significant between-city variance (5.3–8.1%) in PrEP awareness and use underscores the influence of local socio-structural environments on PrEP behavior. Similarly, a U.S. neighborhood-focused study shows geographic clustering of PrEP uptake among MSM [21]. This finding may reflect variations in local health policy implementation, the dynamics of community-based organizations, community acceptance levels, and resource availability that may affect MSM engagement. For instance, a study of multilevel predictors of PrEP use among MSM found disparities between states with different MSM acceptance levels [22]. In this study, willingness to use PrEP showed no significant city-level variation, suggesting that it is primarily a reflective, internally driven stage, less sensitive to contextual factors [23]. This finding was consistent with a study conducted in the U.S., where only PrEP awareness and actual use varied across counties, but not willingness [24]. This distinction suggests that improving structural factors is important in influencing awareness and use, whereas improving willingness needs person-oriented intervention.

Education and income emerged as the most consistent factors associated with PrEP awareness and use, reinforcing global evidence that socioeconomic empowerment facilitates engagement in prevention [12, 25]. Age was also an important factor, with younger MSM more likely to be aware of PrEP, likely reflecting generational differences in social media use, engagement with sexual health networks, and openness to new prevention technologies. Contrary to this, the findings from a study of MSM in the US showed that older age was associated with knowledge of PrEP [26]. This may be due to differences in PrEP-promoting policies and the time elapsed since PrEP rollout began in the two settings. In China, it is a recent development, primarily driven by community-based organizations and social app-based promotions [27], whereas in the US [28], it was rolled out earlier and promoted openly. MSM with receptive or versatile roles were more likely to be aware of or use PrEP than those with exclusively non-insertive roles, consistent with findings from studies in Brazil and West Africa [29, 30]. This might be due to a heightened perceived vulnerability among those MSM who engage in insertive roles.

Lower income was consistently associated with PrEP awareness, willingness, and use, underscoring the economic barrier to biomedical prevention. This finding is corroborated by a study conducted in China, which concluded that the current cost needs to be halved to achieve cost-effectiveness [31]. Similarly, a systematic review found that income is a significant factor in PrEP engagement in low- and middle-income countries [32]. Even when PrEP is subsidized or offered free of charge, the indirect costs, such as clinic visits, time off work, and follow-up testing, have been found to disproportionately burden lower-income individuals [33]. These findings echo Andersen’s emphasis on enabling resources as prerequisites for healthcare utilization. Hence, integrating PrEP into insurance and expanding low-cost community-based services could reduce financial barriers.

Knowledge of a partner’s HIV status was positively associated with awareness, suggesting that relationship-level communication can act as a prevention cue. Similarly, a systematic review and meta-analysis reported that sexual activity with HIV-positive partners was linked to increased PrEP adherence among MSM [34]. Objective behavioral risks, including condomless anal intercourse, multiple sexual partners, and participation in group sex, were strongly linked to PrEP use. Such sexual behaviors were found to increase risk perception among MSM [35]. Engagement in commercial sex was similarly associated with PrEP use, possibly reflecting targeted interventions by health programs. These findings indicate that higher objective HIV risk and perceived need are aligned. However, as reported in other studies among MSM [36], the potential for risk compensation after PrEP initiation may be present, although it cannot be confirmed here due to the study's design, which limits temporal assessment.

Higher income inequality was associated with reduced awareness, consistent with research showing that inequitable resource distribution can limit the reach of health services [37] that might exacerbate disparities in access to information. In addition, HIV prevalence among MSM in the province, a need factor, was another factor strongly associated with awareness. This may be due to a high level of health program activities in areas with a high prevalence of HIV. In addition, many of the high-prevalence areas were included in the HIV PrEP pilot projects, which may have increased awareness in those areas. Interestingly, no contextual variables remained significant in the final multivariable models for PrEP use, despite their role in awareness. This suggests that while structural conditions shape the informational environment, initiating PrEP is more strongly determined by individual-level enabling and need factors.

Living in cities where there is a high provincial HIV prevalence among MSM was associated with a lower willingness to use PrEP despite greater exposure to HIV risk. This paradox suggests that a high epidemiological risk does not automatically translate into higher PrEP uptake intent. It may reflect a disconnect between the objective and perceived needs, possibly driven by stigma, prevention fatigue, or distrust in medical care [38]. In high-prevalence settings, condoms might be more familiar and visibly protective, whereas PrEP’s biomedical mechanism may still be perceived as “only for sick people.” Combined with the documented medical distrust, weak promotion by the healthcare system, and concerns about failure, these perceptions may make PrEP adoption feel riskier in high-prevalence areas.

The concentration of risk behaviors among condom non-users who are unaware of, unwilling to use, or have never used PrEP represents a critical missed opportunity. In Andersen’s model terms, these individuals exhibit a high objective need but lack the enabling resources or perceived vulnerability necessary to engage in prevention. They remain at the intersection of the highest transmission risk and zero protection, continuing to drive new HIV infections despite broader PrEP scale-up efforts. By linking behavioral risk profiles with stages of the PrEP continuum, this finding underscores the public health significance of improving PrEP uptake among high-risk MSM.

This study makes a significant and novel contribution by applying Andersen’s Behavioral Model within a multilevel analytic framework. Leveraging a large, geographically diverse sample and employing GLMM, it provides a robust examination of both individual and contextual influences on PrEP-related outcomes. However, several limitations must be acknowledged. First, the cross-sectional design restricts the ability to draw causal inferences. Second, reliance on self-reported data introduces potential for recall and social desirability bias. Third, although the study incorporated a range of contextual variables, these may not be sufficient to capture local structural or policy-related factors, such as PrEP service availability, municipal health promotion efforts, or healthcare-related stigma, which may influence PrEP engagement. Furthermore, the use of provincial HIV prevalence as a proxy for the city is likely to introduce non-differential misclassification of the contextual exposure. As a result, the association is most likely attenuated. Fourth, the outcome variable ever-use doesn’t qualify as persistent or current use. Finally, restricting the analysis to cities with 30 or more participants may limit generalizability, as smaller cities are underrepresented and city-level variation may not fully capture the broader national context. Future research would benefit from longitudinal designs and the inclusion of health system and policy-level data to better inform targeted interventions and resource allocation.

## Conclusion

This study reveals that the cities where the MSM live influence HIV PrEP awareness and use outcomes. PrEP awareness, willingness, and use are influenced by a range of predisposing, enabling, and need-related factors at both the individual and contextual levels. Contextual predisposing and enabling factors play a stronger role in shaping awareness. In contrast, individual enabling and need factors, particularly income and sexual risk behaviors, are more predictive of actual use. Hence, context is important, but personal agency and resources determine HIV PrEP use among MSM in China. These findings underscore the need for tailored, city-level interventions alongside individual-level support programs. By highlighting the distinct drivers of awareness versus use, the study provides policymakers with actionable evidence to allocate resources efficiently, prioritizing structural interventions to improve knowledge and targeted subsidies to promote uptake. This dual approach is essential to close the PrEP implementation gap and reduce HIV incidence among Chinese MSM.

## Supplementary Information

Additional file 1

## Data Availability

The datasets used and analyzed during the current study are available from the corresponding authors upon reasonable request.

## Abbreviations

PrEP: Pre-exposure prophylaxis
MSM: Men who have sex with men
GLMM: Generalized linear mixed model
*cOR*: Crude odds ratio
*aOR*: Adjusted odds ratio
*CI*: Confidence interval
ICC: Intraclass correlation coefficient
AIC: Akaike information criterion
BIC: Bayesian information criterion
Ʈb: City level random intercept variance
SE: Standard error
HIV: Human immunodeficiency virus
AIDS: Acquired immunodeficiency syndrome
GINI: Gini coefficient, a measure of income inequality
