# Supplementary material for: Where you live matters, but action is personal: insights from multilevel analysis of pre-exposure prophylaxis awareness, willingness, and use among men who have sex with men in China
Source: Infect Dis Poverty. 2026 May 25;15:60. doi: 10.1186/s40249-026-01462-5 (PMC13200295; doi:10.1186/s40249-026-01462-5)
Supplement: Supplementary file 1 — Additional file 1 [file 40249_2026_1462_MOESM1_ESM.docx]

**Where you live matters, but action is personal: insights from multilevel analysis of pre-exposure prophylaxis awareness, willingness, and use among men who have sex with men in China**

**Supplementary material**

**Supplementary Table 1. Crosstabulation of participants’ background characteristics based on PrEP outcomes with the chi-squared test**

| Variable | Category | Heard about  PrEP (*n* = 5041) | | *P* - value | Willing to use PrEP (*n* = 3799) | | *P* - value | Ever used  PrEP (*n* = 4396) | | *P* - value |
| --- | --- | --- | --- | --- | --- | --- | --- | --- | --- | --- |
|  |  | Yes *n* (%) | No |  | Yes *n* (%) | No |  | Yes *n* (%) | No |  |
| Age group | Median, (p25, p75) | 28, (23.59,34) | | **0.003^‡^** | 28.03, (23.5,34) | | 0.64^‡^ | 28, (23.61, 33.65) | | **0.002^‡^** |
| Education level | Junior high & below | 229 (75.8) | 73 (24.2) | **<0.001** | 137 (59.6) | 93 (40.4) | 0.21 | 28 (12.2) | 201 (87.8) | **<0.001** |
|  | High school/Tech sec. | 681 (83.9) | 131 (16.1) |  | 331 (54.5) | 276 (45.5) |  | 65 (9.5) | 616 (90.5) |  |
|  | College/Undergrad | 2965 (88.1) | 401 (11.9) |  | 1506 (58.9) | 1049 (41.1) |  | 380 (12.8) | 2585 (87.2) |  |
|  | Postgraduate or above | 521 (92.9) | 40 (7.1) |  | 243 (59.7) | 168 (40.3) |  | 97 (18.6) | 424 (81.4) |  |
| Occupation type | Not in the labor force | 135 (90.0) | 15 (10.0) | **<0.001** | 65 (58.6) | 46 (41.4) | 0.54 | 20 (14.8) | 115 (85.2) | **0.001** |
|  | Student | 773 (90.2) | 84 (9.8) |  | 375 (56.9) | 284 (43.1) |  | 75 (9.7) | 698 (90.3) |  |
|  | Informal/self-employment | 1494 (84.3) | 279 (15.7) |  | 803 (59.9) | 538 (40.1) |  | 175 (11.7) | 1319 (88.3) |  |
|  | Formal employment | 1994 (88.2) | 267 (11.8) |  | 974 (57.7) | 714 (42.3) |  | 300 (15.0) | 1694 (85.0) |  |
| Monthly income | No stable income | 833 (88.8) | 105 (11.2) | **<0.001** | 410 (56.9) | 311 (43.1) | 0.13 | 81 (9.7) | 752 (90.3) | **<0.001** |
|  | <3000 CYN | 380 (84.4) | 70 (15.6) |  | 179 (52.5) | 162 (47.5) |  | 43 (11.3) | 337 (88.7) |  |
|  | 3000–5000 CYN | 1086 (83.0) | 222 (17.0) |  | 585 (59.9) | 392 (40.1) |  | 116 (10.7) | 970 (87.3) |  |
|  | 5000–8000 CYN | 1039 (87.8) | 145 (12.2) |  | 519 (59.4) | 355 (40.6) |  | 132 (12.7) | 907 (87.3) |  |
|  | >8000 CYN | 1058 (91.1) | 103 (8.9) |  | 524 (59.1) | 362 (40.9) |  | 198 (18.7) | 860 (81.3) |  |
| Commercial sex, ever | No | 4139 (87.0) | 616 (13.0) | 0.16 | 2089 (58.3) | 1496 (41.7) | 0.65 | 510 (12.3) | 3629 (87.7) | **<0.001** |
|  | Yes | 257 (89.9) | 29 (10.1) |  | 128 (59.8) | 86 (40.2) |  | 60 (23.3) | 197 (76.6) |  |
| STI in the past year | No | 4027 (87.1) | 594 (12.9) | 0.67 | 2045 (58.5) | 1450 (41.5) | 0.51 | 500 (12.4) | 3527 (87.6) | **<0.001** |
|  | Yes | 369 (87.9) | 51 (12.1) |  | 172 (56.6) | 132 (43.4) |  | 70 (19.0) | 299 (81.0) |  |
| Number of sex partners (6 months) | 1-5 | 3810 (87.3) | 554 (12.7) | 0.54 | 1922 (58.3) | 1376 (41.7) | 0.93 | 430 (11.3) | 3380 (88.7) | **<0.001** |
|  | 6-10 | 390 (87.4) | 56 (12.6) |  | 187 (58.4) | 133 (41.6) |  | 73 (18.7) | 317 (81.3) |  |
|  | >10 | 196 (84.8) | 35 (15.2) |  | 108 (59.7) | 73 (40.3) |  | 67 (34.2) | 129 (65.8) |  |
| Group sex (6 months) | No | 3812 (87.0) | 571 (13.0) | 0.21 | 1926 (58.3) | 1378 (41.7) | 0.83 | 417 (10.9) | 3395 (89.1) | **<0.001** |
|  | Yes | 584 (88.8) | 74 (11.2) |  | 291 (58.8) | 204 (41.2) |  | 153 (26.2) | 431 (73.8) |  |
| Sex role | Receptive | 1443 (89.2) | 174 (10.8) | <**0.001** | 705 (56.5) | 543 (43.5) | 0.34 | 204 (14.1) | 1239 (85.9) | **0.001** |
|  | Versatile | 1011 (88.1) | 137 (11.9) |  | 496 (58.1) | 357 (41.9) |  | 154 (15.2) | 857 (84.8) |  |
|  | Insertive | 1653 (85.9) | 271 (14.1) |  | 863 (59.9) | 578 (40.1) |  | 190 (11.5) | 1463 (88.5) |  |
|  | Oral only | 289 (82.1) | 63 (17.9) |  | 153 (59.5) | 104 (40.5) |  | 22 (7.6) | 267 (92.4) |  |
| Condom use at last anal sex | No | 809 (86.9) | 122 (13.1) | 0.47 | 412 (58.1) | 297 (41.9) | 0.92 | 158 (19.5) | 651 (80.5) | **<0.001** |
|  | Yes | 3298 (87.8) | 460 (12.2) |  | 1652 (58.3) | 1181 (41.7) |  | 390 (11.8) | 2908 (88.2) |  |
| Partner’s HIV status (past 6 months) | Didn’t know all | 702 (80.9) | 166 (19.1) | **<0.001** | 383 (57.9) | 279 (42.1) | 0.31 | 92 (13.1) | 610 (86.9) | **<0.001** |
|  | Partially knew | 1599 (88.8) | 201 (11.2) |  | 813 (60.0) | 542 (40.0) |  | 251 (15.7) | 1348 (84.3) |  |
|  | Knew all | 2095 (88.3) | 278 (11.7) |  | 1021 (57.3) | 761 (42.7) |  | 227 (10.8) | 1868 (89.2) |  |

**‡ Mann-Whitney test**

**Supplementary Table 2. Factors Associated with Ever Hearing of PrEP GLMM: Four models**

| Variable | *cOR* [95% *CI*] | Individual level *aOR1* [95% *CI*] | Contextual level *aOR2* [95% *CI*] | Full model  *aOR3* [95% *CI*] |
| --- | --- | --- | --- | --- |
| Age | 0.97 [0.96–0.98] *** | 0.98 [0.97-0.99] * |  | 0.98 [0.97-0.99] * |
| Education (Ref: Graduate+) | | | | |
| Junior high & below | 0.27 [0.18–0.42] *** | 0.36 [0.23-0.58] *** |  | 0.34 [0.22–0.54] *** |
| High school/Tech sec. | 0.47 [0.32–0.79] *** | 0.58 [0.39-0.87] ** |  | 0.56 [0.38–0.84] ** |
| College/Undergrad | 0.62 [0.44–0.87] ** | 0.67 [0.47-0.95]* |  | 0.64 [0.45-0.91] * |
| Occupation (Ref: Formal employment) | | | | |
| Informal/Self-employed | 0.79 [0.69–0.95] * |  |  | – |
| Income (Ref: >8000 CYN) | | | | |
| <3000 CYN | 0.59 [0.42–0.82] ** | 0.67 [0.46-0.98] * |  | 0.67 [0.47–0.95] * |
| 3000–5000 CYN | 0.53 [0.41–0.69] *** | 0.61 [0.46-0.79] *** |  | 0.61 [0.46–0.80] *** |
| 5000–8000 CYN | 0.75 [0.57-0.99] * |  |  |  |
| Sex role (Ref: No insertive) | | | | |
| Receptive only | 1.79 [1.3–2.46] *** | 1.55 [1.11-2.15] ** |  | 1.61 [1.16-2.25] ** |
| Versatile | 1.7 [1.22–2.37] ** | 1.56 [1.11-2.19] * |  | 1.61 [1.14-2.27] ** |
| Insertive | 1.39 [1.02-1.89] * |  |  |  |
| Condom use (last sex) (Ref: Yes) | | | | |
| No | 1.75 [1.42-2.16] *** |  |  |  |
| HIV status of partner (Ref: Knew all) | | | | |
| Didn't know all | 0.53 [0.43–0.66] *** | 0.55 [0.44 – 0.69] *** | – | 0.57 [0.45–0.71] *** |
| GINI index | 0.04 [0.005–0.37] ** |  | 0.03 [0.003–0.34] ** | 0.03 [0.003–0.35] ** |
| MSM HIV rate (province) | 1.07 [1.04–1.11] *** |  | 1.07 [1.04–1.11] *** | 1.08 [1.04–1.13] *** |
| Health professionals (per capita) | 1.85 [1.21–2.82] ** |  | – | – |
| Health expenditure (per capita) | 1.05 [1.03–1.08] *** |  | 1.03 [1.01-1.06] * | – |

Note: *cOR* = crude odds ratio; *aOR1* = individual‑level adjusted odds ratio; *aOR2* = contextual‑level adjusted odds ratio; *aOR3* = full model (individual + contextual) adjusted odds ratio. All models are mixed‑effects logistic regressions. 95% confidence intervals (*CI*) are shown in brackets. Significance: *** $P<0.001$, ** $P<0.01$, * $P<0.05$. A dash (—) indicates that the variable was not included in that model. Reference categories: Education (Graduate+), Occupation (Formal employment), Income (>8000 CYN), Sex role (No insertive), Condom use (Yes), HIV status of partner (Knew all). CYN =. Chinese yuan. GINI = Greater income inequality.

**Supplementary Table 3. Factors associated with ever-using PrEP**

| Variable | *cOR* [95% *CI*] | Individual level  *aOR1* [95% *CI*] | Contextual level *aOR2*  [95% *CI*] |  |
| --- | --- | --- | --- | --- |
| Age | 1.01 [1.001–1.024] * | – |  |  |
| STI diagnosis (Ref: Yes) | 0.65 [0.49–0.86] ** | – |  |  |
| Education (Ref: Graduate+) | | | | |
| High school/Tech sec. | 0.49 [0.34–0.71] *** | 0.56 [0.39–0.84] ** |  |  |
| College/Undergrad | 0.64 [0.51–0.83] ** | 0.75 [0.57-0.99] * |  |  |
| Occupation (Ref: Formal employment) | | | | |
| Student | 0.62 [0.47–0.82] ** |  |  |  |
| Informal/Self-employed | 0.76 [0.62–0.93] * |  |  |  |
| Income (Ref: >8000 CYN) | | | | |
| No stable income | 0.39 [0.28–0.54] *** |  |  |  |
| <3000 CYN | 0.45 [0.29–0.69] *** |  |  |  |
| 3000–5000 CYN | 0.52 [0.39–0.70] *** | 0.65 [0.49–0.88] ** |  |  |
| 5000–8000 CYN | 0.58 [0.45–0.76] *** | 0.75 [0.58–0.98] * |  |  |
| Sex role (Ref: No insertive) | | | | |
| Receptive only | 2.02 [1.26–3.21] ** | 1.36 [1.09–1.71] ** |  |  |
| Versatile | 2.21 [1.37-3.54] ** | 1.31 [1.03–1.68] * |  |  |
| Insertive | 1.61 [1.01–2.56] * | – |  |  |
| Involvement in commercial sex work (Ref: Yes) | | | | |
| No | 0.47 [0.34-0.64] *** | 0.61 [0.43-0.86] ** |  |  |
| Condom use (last sex) (Ref: Yes) | 1.81 [1.45–2.26] *** | 1.62 [1.31–2.03] *** |  |  |
| Number of sex partners (Ref: >10) | | | | |
| 1–5 | 0.24 [0.17–0.33] *** | 0.47 [0.32–0.69] *** |  |  |
| 6–10 | 0.44 [0.29–0.66] *** | 0.59 [0.38–0.91] * |  |  |
| Group sex (Ref: Yes) | | | | |
| No | 0.32 [0.26–0.41] *** | 0.49 [0.37–0.63] *** |  |  |
| HIV status of partner (Ref: Knew all) | | | | |
| Didn’t know all | 1.22 [0.94–1.59] |  |  |  |
| Partially knew | 1.54 [1.26-1.88] *** |  |  |  |
| Number of PrEP info sources (Ref: 5) | | | | |
| 1 | 0.67 [0.47–0.95] * |  |  |  |
| 2 | 0.55 [0.38–0.78] ** |  |  |  |
| 3 | 0.58 [0.41–0.84] ** |  |  |  |
| PrEP info source (Ref: Formal source) | | | | |
| Internet source | 0.62[0.47–0.81] *** |  |  |  |

Note: *cOR* = crude odds ratio; *aOR1* = individual‑level adjusted odds ratio; *aOR2* = contextual‑level adjusted odds ratio; *aOR3* = full model (individual + contextual) adjusted odds ratio. All models are mixed‑effects logistic regressions. 95% confidence intervals (*CI*) are shown in brackets. Significance: *** $P<0.001$, ** $P<0.01$, * $P<0.05$. A dash (—) indicates that the variable was not included in that model. Reference categories: Education (Graduate+), Income (>8000 yuan), Sex role (No insertive), Involvement in commercial sex work (Yes), Condom use (Yes), Number of sex partners (>10), Group sex (Yes), HIV status of partner (Knew all). “Number of PrEP info sources” is compared to 5 sources; “PrEP info source” contrasts Internet sources with formal sources (e.g., health professionals, community organizations). CYN =. Chinese yuan.

**Supplementary Table 4. Determinants of Willingness to Use PrEP (Binary Logistic Regression)**

| Variables | *cOR* | 95% *CI* | *aOR* | 95% *CI* |
| --- | --- | --- | --- | --- |
| Monthly Income (ref: >8000 CYN) | | |  | |
| No stable income | 0.91 | [0.74 – 1.11] | 0.92 | [0.75 – 1.12] |
| <3000 CYN | 0.76 | [0.59 – 0.98] *** | 0.75 | [0.60 – 0.99] |
| 3000–5000 CYN | 1.03 | [0.85 –1.24] | 1.03 | [0.85 –1.24] |
| 5000-8000 CYN | 0.01 | [0.83 –1.22] | 1.02 | [0.84 – 1.23] |
| HIV status of partner (Ref: Knew all) | | |  | |
| Partially knew | 1.12 | [1.02, – 1.22] *** | 1.028 | [0.85 – 1.23] |
| Didn’t know all | 1.04 | [0.55 –1.94] | 1.11 | [0.96 – 1.28] |
| HIV prevalence, MSM | 0.96 | [0.94 – 0.98] ** | 0.96 | [0.94 – 0.98] ** |
| Healthcare Expenditure | 1.02 | [1.01 – 1.03] *** | 1.02 | [1.01–1.03] *** |

Note: *cOR* = crude odds ratio; *aOR* = adjusted odds ratio (full model with all listed variables). 95% *CI* are shown in brackets. Significance: *** $P<0.001$, ** $P<0.01$, * $P<0.05$. The p‑value column corresponds to the Wald test for each odds ratio. Reference categories: Monthly Income (>8000 CYN), HIV status of partner (Knew all). “HIV prevalence, MSM” is the provincial HIV prevalence among men who have sex with men; “Healthcare Expenditure” is per capita public health expenditure (log‑transformed in analysis). All other variables (e.g., age, education) were tested but not retained because they were non‑significant in both crude and adjusted models; only variables with at least one significant estimate are shown. CYN =. Chinese yuan.

**Supplementary Table 4. Geographic distribution of the 42 included cities by province and major geographical region of China**

| City Name | Province / Equivalent Administrative Unit | Major Geographical Region |
| --- | --- | --- |
| Baoding | Hebei Province | North China |
| Beijing | Beijing Municipality | North China |
| Changchun | Jilin Province | Northeast China |
| Changsha | Hunan Province | Central China |
| Changzhi | Shanxi Province | North China |
| Chengdu | Sichuan Province | Southwest China |
| Chongqing | Chongqing Municipality | Southwest China |
| Deyang | Sichuan Province | Southwest China |
| Fuzhou | Fujian Province | East China |
| Guangzhou | Guangdong Province | South China |
| Haikou | Hainan Province | South China |
| Handan | Hebei Province | North China |
| Hangzhou | Zhejiang Province | East China |
| Hefei | Anhui Province | East China |
| Hohhot | Inner Mongolia Autonomous Region | North China |
| Huizhou | Guangdong Province | South China |
| Huzhou | Zhejiang Province | East China |
| Jixi | Heilongjiang Province | Northeast China |
| Kunming | Yunnan Province | Southwest China |
| Lanzhou | Gansu Province | Northwest China |
| Mianyang | Sichuan Province | Southwest China |
| Mudanjian | Heilongjiang Province | Northeast China |
| Nanchang | Jiangxi Province | East China |
| Nanjing | Jiangsu Province | East China |
| Qingdao | Shandong Province | East China |
| Shanghai | Shanghai Municipality | East China |
| Shaoxing | Zhejiang Province | East China |
| Shenyang | Liaoning Province | Northeast China |
| Shenzhen | Guangdong Province | South China |
| Shijiazhuang | Hebei Province | North China |
| Siping | Jilin Province | Northeast China |
| Suzhou | Jiangsu Province | East China |
| Tianjin | Tianjin Municipality | North China |
| Urumuqi | Xinjiang Uygur Autonomous Region | Northwest China |
| Wuhan | Hubei Province | Central China |
| Wuxi | Jiangsu Province | East China |
| Xian | Shaanxi Province | Northwest China |
| Yibing | Sichuan Province | Southwest China |
| Yinzhou | Ningxia Hui Autonomous Region | Northwest China |
| Yuxi | Yunnan Province | Southwest China |
| Zhengzhou | Henan Province | Central China |
| Zunyi | Guizhou Province | Southwest China |
